# Predictive value of specific IgE for clinical peanut allergy in children: relationship with eczema, asthma, and setting (primary or secondary care)

**DOI:** 10.1186/2045-7022-3-34

**Published:** 2013-10-10

**Authors:** Wilma J van Veen, Lambert D Dikkeschei, Graham Roberts, Paul LP Brand

**Affiliations:** 1 Princess Amalia Children’s Centre, Isala hospital, PO Box 10400, 8000 GK, Zwolle, the Netherlands; 2 Clinical Laboratory, Isala hospital, Zwolle, the Netherlands; 3 Clinical and Experimental Sciences Academic Unit, Faculty of Medicine, University of Southampton, Southampton, UK; 4 David Hide Asthma and Allergy Research Centre, St Mary’s Hospital, Newport, Isle of Wight, UK; 5 UMCG Postgraduate School of Medicine, University Medical Centre, Groningen, the Netherlands

**Keywords:** Peanut allergy, Peanut-specific IgE, Peanut sensitization, Eczema, Asthma, Children, Teenagers

## Abstract

The usefulness of peanut specific IgE levels for diagnosing peanut allergy has not been studied in primary and secondary care where most cases of suspected peanut allergy are being evaluated. We aimed to determine the relationship between peanut-specific IgE levels and clinical peanut allergy in peanut-sensitized children and how this was influenced by eczema, asthma and clinical setting (primary or secondary care). We enrolled 280 children (0–18 years) who tested positive for peanut-specific IgE (> 0.35 kU/L) requested by primary and secondary physicians. We used predefined criteria to classify participants into three groups: peanut allergy, no peanut allergy, or possible peanut allergy, based on responses to a validated questionnaire, a detailed food history, and results of oral food challenges.

Fifty-two participants (18.6%) were classified as peanut allergy, 190 (67.9%) as no peanut allergy, and 38 (13.6%) as possible peanut allergy. The association between peanut-specific IgE levels and peanut allergy was significant but weak (OR 1.46 for a 10.0 kU/L increase in peanut-specific IgE, 95% CI 1.28-1.67). Eczema was the strongest risk factor for peanut allergy (aOR 3.33, 95% CI 1.07-10.35), adjusted for demographic and clinical characteristics. Asthma was not significantly related to peanut allergy (aOR 1.93, 95% CI 0.90-4.13). Peanut allergy was less likely in primary than in secondary care participants (OR 0.46, 95% CI 0.25-0.86), at all levels of peanut-specific IgE.

The relationship between peanut-specific IgE and peanut allergy in children is weak, is strongly dependent on eczema, and is weaker in primary compared to secondary care. This limits the usefulness of peanut-specific IgE levels in the diagnosis of peanut allergy in children.

## Introduction

Although the double-blind placebo-controlled food challenge (DBPCFC) is the gold standard for diagnosing peanut allergy [1], its use in daily practice is limited because it is time consuming, expensive, and not available in all hospitals. In practice, the diagnosis of peanut allergy is usually based on a suggestive clinical history, together with evidence of allergic sensitization to whole peanut allergen [2-4]. There are however no universally agreed criteria for a suggestive clinical history. For example, are both objective symptoms, such as urticaria or vomiting, and subjective ones, such as abdominal pain, mouth and tongue tingling diagnostic; should symptoms always occur reproducibly after each exposure and remain absent without exposure to the allergen; and how close should the temporal relationship between exposure and symptoms be? The lack of uniformity of criteria for a suggestive clinical history may lead to over-and underdiagnosis of peanut allergy [4]. Parental suspicion of peanut allergy in their child is unreliable, with parent-suspected peanut allergy being much more common than peanut allergy confirmed by DBPCFC [5].

High levels of peanut-specific IgE are taken to indicate clinical allergy to peanut [6]. Unfortunately, the cut-off levels of peanut-specific IgE above which >95% of children are clinically allergic to peanut vary from 15 to 57 kU/l in different studies [6-9]. This is likely to result from differences in study populations and food challenge protocols. As peanut-specific IgE levels have only been studied in general population samples or in tertiary care food allergy centres, it’s unclear how useful they are in predicting clinical peanut allergy in children seen in primary and secondary care, where most cases of suspected peanut allergy are evaluated.

As peanut sensitization is strongly related to loss-of-function variations in the filaggrin gene found in eczema [10] and to asthma [11], the relationship between peanut sensitization and peanut allergy may be confounded by eczema and asthma. To our knowledge, this has never been studied to date.

The purpose of this study was to determine the relationship between the level of peanut-specific IgE and clinical peanut allergy in peanut-sensitized. Additionally, we aimed to assess the confounding influence of eczema and asthma, and of setting (primary or secondary care) on this relationship.

## Methods

### Study population

The study population included all 427 children (aged 0–18 years) tested positive to peanut-specific IgE (> 0.35 kU/L) in our laboratory between 2003 and 2010. In the Netherlands, children with suspected allergies are first seen by general practitioners (GPs), and can only be assessed by a paediatrician after referral by their GP. Paediatricians in the Netherlands are hospital-based and provide secondary or tertiary paediatric care. Specific IgE testing is the routine method of allergy testing by GPs and paediatricians in the Netherlands [12]. Our clinical laboratory is the only laboratory performing specific IgE testing in the catchment area of our hospital, both for hospital-based medical specialists and for GPs. The ImmunoCap system (Thermo Fisher, Uppsala, Sweden) was used for all specific IgE assessments throughout the study period [9].

In 2011, all these 427 subjects were invited to participate in the present study, which was approved by the hospital’s ethical review board. Parents, and where appropriate participants, provided written informed consent.

### Clinical assessment of peanut allergy

All 427 participants and their parents were mailed a validated questionnaire (Food Allergy Quality of Life Questionnaire) [13] to obtain information on exposure to peanut and symptoms associated with it. Children who reported recent ingestion of peanut in the last month without a reaction were considered to not have peanut allergy. Children who reported a reaction on exposure to peanuts were invited for a detailed food allergy history. This consisted of a comprehensive review of symptoms on exposure to foods containing peanut, and about the occurrence of these symptoms without exposure to peanut. Based on previous work defining positive food challenges [14] and diagnosing peanut allergy by history [15], we used predefined specific history criteria to define participants as having or not having peanut allergy (Table 1).

**Table 1:** **Criteria for diagnosis or exclusion of clinical peanut allergy [**[14]**]**

Peanut allergy	Reproducible, objective symptoms (vomiting, urticaria/angio-oedema, wheeze, anaphylaxis), within a plausible timeframe after recent exposure to a relevant quantity of peanut; *and* never experiencing these symptoms without eating peanut
Possible peanut allergy	- No reported exposure to a relevant quantity of peanut
	- Exclusively subjective symptoms
	- Not clearly reproducible symptoms
No peanut allergy	- Objective symptoms without a clear and consistent relationship to reported peanut exposure, *or*
	- Reported recent exposure to to a relevant quantity of peanut without reproducible symptoms, *and*
	- Another plausible cause for the patient’s symptoms

Participants with reproducible objective symptoms within a reasonable timeframe after each exposure to peanut and no such symptoms during avoidance of peanut were classified as having peanut allergy. Participants without a history of anaphylaxis or severe asthma who did not meet any of these criteria were encouraged to reintroduce peanut into their diet. These patients were followed up by telephone and clinic visits. If peanut was reintroduced without symptoms, they were defined as not having peanut allergy. Participants who developed objective symptoms upon exposure at home were defined as having peanut allergy. When peanut allergy could not be confirmed or rejected using this approach, participants were offered a DBPCFC (using validated recipes for peanut hidden in cookies, as previously described [16]) in our clinic. Participants with an unclear history who declined a DBPCFC were defined as having possible peanut allergy. This clinical assessment of peanut allergy was made without knowledge of participants’ level of peanut-specific IgE.

### Assessment of asthma

Asthma symptoms were recorded using the ISAAC questionnaire [17]. Children were defined as having asthma if they had a doctor’s diagnosis of asthma ever, and had experienced an episode of wheeze or had used bronchodilators or daily maintenance medication in the last 12 months. Participants invited for a detailed food allergy history completed the Dutch translation of the Asthma Control Questionnaire (ACQ) [18], and participants aged 6 years and older performed spirometry before and after inhalation of 400 ug of salbutamol as previously described [19]. Well-controlled asthma was defined as ACQ <1.0 and an FEV_1_ of ≥ 80% of predicted.

### Assessment of eczema

Eczema was defined as a positive response to: has the child ever been diagnosed with eczema by a doctor, and has the child had an itchy skin condition and generally dry skin with onset before the age of 2 years, with flexural involvement? [20].

### Statistical analysis

Data were analysed using SPSS19 for Windows. Due to the skewed distributions even after logarithmic transformation, peanut-specific IgE was analysed by non-parametric methods (Mann–Whitney U test). Chi-squared tests were used to determine the relation between peanut allergy and clinical characteristics. Multiple logistic regression was used to examine the association between peanut allergy and levels of peanut-specific IgE, and to adjust this for potential confounding by asthma, eczema and clinical setting.

## Results

Of the 427 participants, 280 (65%) were assessed in the study. Clinical characteristics of these participants are presented in Table 2. There were no significant differences in age, gender, setting, and specific IgE levels between those who participated in the study assessment and those who declined participation (Table 2). The median (interquartile range [IQR]) duration between measurement of peanut-specific IgE and clinical assessment of peanut allergy was 4.3 (4.1-6.0) years.

**Table 2:** Characteristics of study population

	**Participants**	**Range IQR**	**Non-participants**	**Range IQR**	**p-value**
	**(n=280)**		**(n=147)**		
	**n (%) / median**		**n (%) / median**		
*Male gender*	183 (65.4)		89 (61.0)		0.370
*Age at IgE measurement (years)*	6.9	0.3-18.0	6.5	0.5-18.0	0.876
		3.5-11.4		3.3-12.2	
*Age at study participation*	11.4	2.5-24.1	11.6	2.0-24.7	0.501
		7.7-16.0		8.0-16.9	
*Primary care*	176 (62.9)		93 (63.3)		0.934
*Level of peanut-specific IgE (kU/l)*	2.35	0.4-100.0	2.95	0.4-100.0	0.716
		0.9-11.5		0.9-9.7	
*Level of total IgE (kU/L)*	426	6-5000	414	17-4755	0.809
		151-1020		163-1061	
*Atopic disease in history*	266 (95.0)				
- *Eczema*	213 (76.9)				
- *Asthma*	139 (49.6)				
- *Allergic rhinitis*	179 (67.0)				
*Family history of allergic disease*	205 (89.9)				

P values represent results of chi squared tests for proportions and Mann–Whitney U test for comparison of medians.

### Peanut allergy

**Figure 1:**
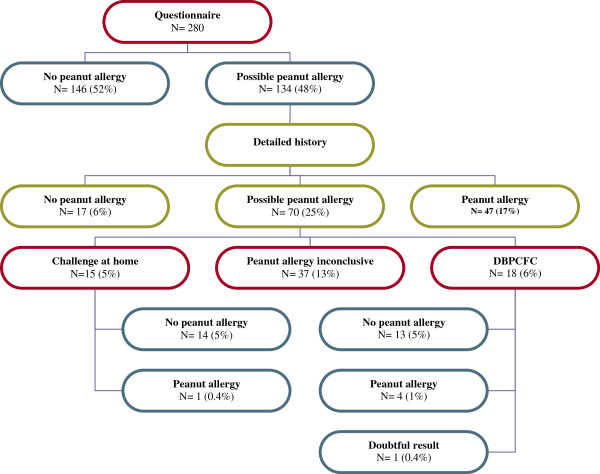
Study flowchart and classification of participants.

The assessment of study participants is described in Figure 1. A total of 52 participants (18.6%) were defined as having peanut allergy (15 on the basis of a positive DBPCFC before the study, 14 on the basis of a positive DBPCFC during the study, and 23 as per criteria in Table 1). Thirteen children with peanut allergy (25.0%) reported symptoms in one organ system (most commonly skin or gastrointestinal tract), and 22 (42.3%) had symptoms in two organ systems. Seventeen children (32.7%) reported respiratory symptoms after exposure to peanut indicating anaphylaxis. Peanut allergy was excluded in 190 participants (67.9%). A total of 38 (13.6%) were defined as having possible peanut allergy on the basis of the study criteria (Table 1).

### Association between peanut-specific IgE and peanut allergy

**Figure 2:**
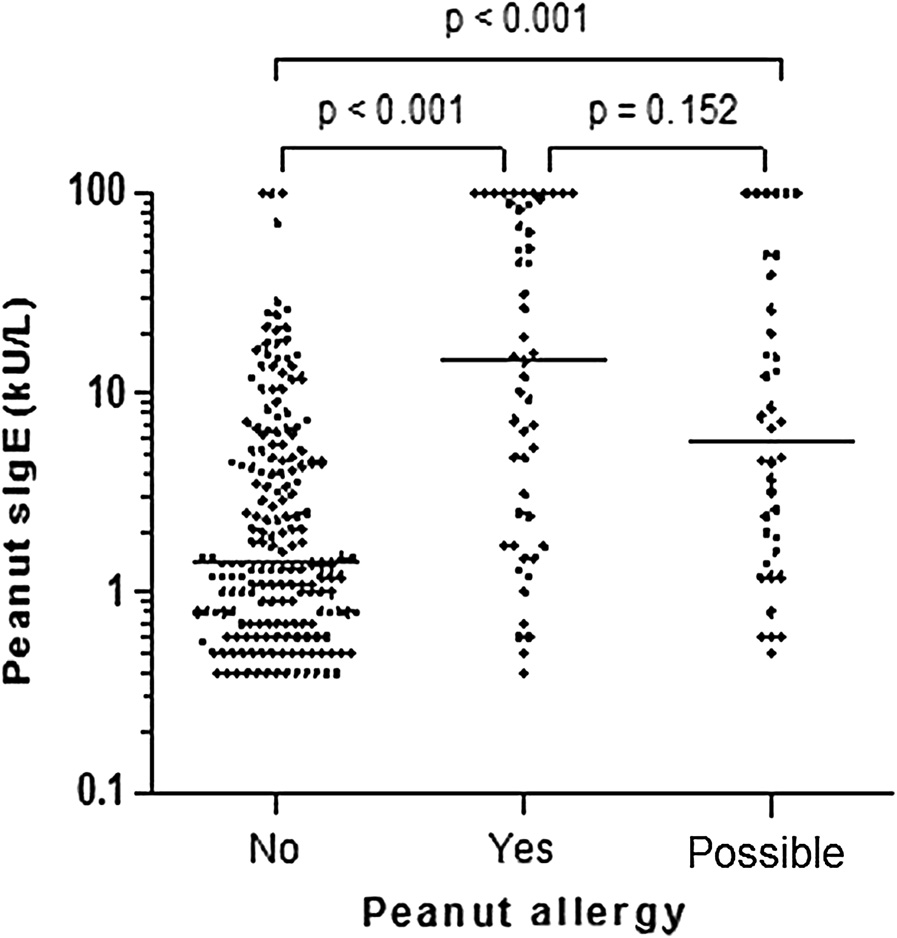
**Level of peanut-specific IgE (sIgE) in children with peanut allergy, no peanut allergy, and possible peanut allergy.** P values represent results of Mann–Whitney U tests.

Participants with peanut allergy had higher peanut-specific IgE levels than those who did not have peanut allergy (p<0.001, Table 3). There was large overlap though in individual peanut-specific IgE levels between participants with peanut allergy, possible peanut allergy, and no peanut allergy (Figure 2). Subjects with possible peanut allergy were excluded from further analyses of the association between peanut-specific IgE and peanut allergy. The likelihood of peanut allergy was 14% at the median level of peanut-specific IgE (2.35 kU/L), and 50% at a peanut-specific IgE level of 51.0 kU/L. The highest probability of peanut allergy was 87% at the highest level of peanut-specific IgE (>100 kU/l).

**Table 3:** Characteristics of children with and without peanut allergy

	**Peanut allergy**	**No peanut allergy**	**p-value**
	**n=52**	**n=190**	
	**n (%) /**	**n (%) /**	
	**median (IQR)**	**median (IQR)**	
*Male sex*	33 (63.5)	124 (65.3)	0.809
*Age at IgE measurement (years)*	5.8 (2.8-12.1)	6.9 (3.8-11.4)	0.506
*Primary care*	25 (48.0%)	127 (66.7%)	0.013
*Level of peanut-specific IgE (kU/l)*	14.8 (1.9-88.5)	1.4 (0.7-5.2)	<0.001
*Level of total IgE (kU/L)*	312 (112–1044)	553 (172–1138)	0.427
*History of atopic disease*	49 (94.2)	181 (95.3)	0.804
- *Eczema*	46 (90.2)	134 (70.5)	0.004
- *Asthma*	32 (61.5)	85 (44.7)	0.032
- *Allergic rhinitis*	29 (58.0)	128 (70.7)	0.088
*Family history of allergy*	41 (93.2)	136 (88.3)	0.355
Good asthma control*	8 (72.7)	15 (75.0)	0.890

* ACQ < 1.0 and FEV1 > 80% predicted.

P values represent results of chi squared tests for proportions and Mann–Whitney U test for comparison of medians.

**Figure 3:**
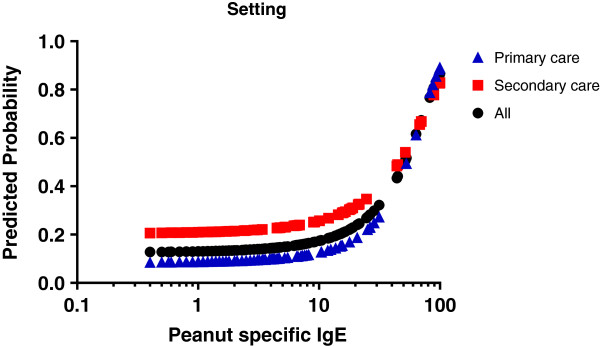
Predicted probability of peanut allergy (logistic regression model) at each given peanut-specific IgE level (sIgE).

The highest likelihood ratio of a positive peanut-specific IgE test for peanut allergy was 16.3 (sensitivity 42%, specificity 97%, positive predictive value [PPV] 79%, negative predictive value [NPV] 86%) at a level of 30.0 kU/L [21]. The lowest likelihood ratio of a negative test was 0.2 (sensitivity 96%, specificity 15%, PPV 24%, NPV 79%) at 0.6 kU/L. The relationship between peanut-specific IgE levels and peanut allergy differed between primary and secondary care participants, with higher probability of peanut allergy at all levels of peanut-specific IgE (Figure 3).

### Association of peanut allergy with demographic and clinical characteristics

The relationship between peanut allergy and demographic and clinical characteristics is presented in Table 4. Eczema was strongly related to peanut allergy (odds ratio [OR] 3.20, 95% CI 1.30-7.93), and remained highly significant after adjustment for age, gender, other atopic diseases, setting, and level of peanut-specific IgE in multiple logistic regression analysis (adjusted OR [aOR] 3.33, 95% CI 1.07-10.35). In this multiple logistic regression model, eczema was a stronger risk factor for peanut allergy than peanut-specific IgE levels (aOR for a 10-kU/L rise 1.45, 95% CI 1.27-1.66). Of all the 213 children with eczema, 46 (22%) had peanut allergy, as compared to 6 (9%) of children without eczema (p=0.009).

**Table 4:** Predictors of clinical peanut allergy, examined in univariate analyses and in multiple logistic regression analysis

**Variable**	**Univariate analysis**		**Multiple logistic regression analysis**	
	***OR***	***95% CI***	***OR***	***95% CI***
*Male gender*	0.92	0.49-1.75	1.03	0.47-2.25
*Age (years)*	1.00	1.00-1.00	1.00	1.00-1.00
*Peanut-specific IgE (10 kU/L)*	1.46	1.28-1.67	1.45	1.27-1.66
*Asthma*	1.98	1.06-3.70	1.93	0.90-4.13
*Eczema*	3.20	1.30-7.93	3.33	1.07-10.35
*Rhinitis*	0.58	0.30-1.09	0.82	0.35-1.89
*Primary care*	0.46	0.25-0.86	0.59	0.30-1.16

Asthma was more common in children with peanut allergy in univariate analysis, but this difference was no longer significant after adjustment for the other variables in the multiple logistic regression model (Table 4). Most children had well controlled asthma (95/139, 68%); there was no association of asthma control to either peanut-specific IgE (p=0.978) or peanut allergy (p=0.890). Children with asthma were no more likely to have reported an anaphylactic reaction to peanut (13/32, 40.6%) than children without asthma (4/20, 20%, p=0.242).

Children in primary care were less likely to have peanut allergy (16%) than those in secondary care (30%) (OR 0.46, 95% CI 0.25-0.86). This remained significant after adjustment for age and gender (aOR 0.47, 95% CI 0.25-0.87), but became non significant after entering presence of atopic diseases and peanut-specific IgE levels into the model (Table 4).

## Discussion

This study shows that the relationship between peanut-specific IgE and peanut allergy is significantly and strongly influenced by the presence of eczema, and differs between children in primary and secondary care. Eczema was a stronger risk factor for clinical peanut allergy than the level of peanut-specific IgE (Table 4). Peanut allergy was more likely in secondary than in primary care, at each level of peanut-specific IgE.

In our study, the proportion of peanut sensitized participants who were defined as having peanut allergy was smaller (Figure 1), and the predictive value of peanut-specific IgE levels for clinical peanut allergy weaker (Figure 3) than in previous research, where peanut allergy could be predicted with 95% probability at peanut-specific IgE cutoff levels between 13.0 kU/L and 57 kU/L, respectively [6-9]. In our population, a predicted probability of 95% was not even achieved at the highest level of peanut-specific IgE (>100 kU/L)as 3 of the 13 children with this high sensitization level were not peanut allergic. This variability in the predictive value of peanut-specific IgE levels for clinical peanut allergy is likely to be due to differences in study populations and definitions of peanut allergy. Our results indicate that the usefulness of peanut-specific IgE levels in diagnosing peanut allergy depends on the presence of eczema and the healthcare setting.

To our knowledge, this is the first study to show that the relationship between peanut-specific IgE and peanut allergy is influenced by a history of eczema. Even after adjustment for age, gender, presence of rhinitis and asthma, and the degree of sensitization to peanut, participants with a history of eczema were three times more likely to have peanut allergy than children without eczema (Table 4). Eczema has been identified as a significant risk factor for peanut allergy [22], and the filaggrin mutations often seen with eczema represent a significant risk factor for IgE-mediated peanut allergy [10]. Results of longitudinal population studies show that eczema precedes peanut sensitization in the majority of patients [23]. These observations suggest that epithelial barrier dysfunction plays a major role in the development of peanut allergy, and that the presence or a history of eczema is a strong marker of this risk factor. We could not confirm the association between asthma control and peanut allergy observed previously [24]. Most previous studies used peanut sensitization as the marker for peanut allergy. We previously showed that peanut sensitization is strongly associated with polysensitization [25]. We hypothesize, therefore, that the association between poorly controlled asthma and peanut allergy is largely explained by the presence of polysensitization, including sensitization to peanut. Our results suggest that clinical peanut allergy is not associated with poorly controlled asthma. In most clinical guidelines, the use of peanut-specific IgE is recommended as a useful part of the diagnostic evaluation of potential peanut allergy [1,2]. In our population, the relationship between peanut-specific IgE and peanut allergy was dependent on eczema, and there was large overlap in peanut-specific IgE values between children with and without peanut allergy (Figure 2). Our results support the view of The Dutch College of General Practitioners that peanut-specific IgE have limited value in the diagnostic workup of peanut allergy [12].

The clinical history is key to the diagnosis of peanut allergy [26]. The strict history criteria that we used (Table 1) were derived from studies on the interpretation of DBPCFC results. Application of these criteria may help clinicians to avoid excessive and unnecessary avoidance of peanut, which contributes to improving quality of life [27]. We did not observe any severe allergic reactions to reintroduction of peanut into the child’s diet using this approach.

The main strengths of our study include the relatively large number of participants who were investigated in primary and secondary care, a population that is under represented in studies. The main weaknesses include the low participation rate and the time lag between peanut-specific IgE assessment and clinical assessment. As the sample studied was representative of the root population referred to the laboratory for specific IgE testing, selection bias is unlikely. The median time lag between the assessments of peanut-specific IgE levels and of peanut allergy was more than 4 years. Although peanut-specific IgE levels may have changed during this time period, the available evidence suggests that peanut peanut allergy and peanut sensitization in children are usually persistent [28]. The 4-year time lag is therefore unlikely to have had a major influence on our results. An additional limitation of our study is that the reason for specific IgE assessments (allergy screening or specific testing for suspected peanut allergy) was not recorded. This may have differed between primary and secondary care. A final limitation is that we did not perform component resolved diagnostics or DBPFCFCs for peanut in all children in our cohort. This, however, reflects current paediatric allergy practice [2].

In conclusion, this study shows that the relationship between peanut-specific IgE and clinical peanut allergy is strongly influenced by the presence of eczema, and differs between primary and secondary care. This limits the usefulness of peanut senistization in the diagnosis of clinical peanut allergy in children.

## Competing interests

The authors declare that they have no competing interests.

## Authors’ contributions

WV collected and analysed study data and wrote the initial draft of the manuscript; LD supervised specific IgE analyses, contributed to design of the study, and edited the manuscript; GR contributed to data analysis and interpretation, and edited the manuscript; PB designed the study, supervised data collection and analysis, and edited the manuscript. All authors approve of the manuscript submitted herewith.
